# Supplementary material for: Comparative multiomics analysis of cell physiological state after culture in a basket bioreactor
Source: Sci Rep. 2022 Nov 23;12:20161. doi: 10.1038/s41598-022-24687-4 (PMC9686226; doi:10.1038/s41598-022-24687-4)
Supplement: Supplementary file 1 — Supplementary Information 1. [file 41598_2022_24687_MOESM1_ESM.zip › raw data/Transcriptomic raw data/7.AS/2.dasplot/TE1vsTC1/A3SS/9_CNKSR2_NW_023666056.1_21941001_21941547_+@NW_023666056.1_21971162_21978591_+@NW_023666056.1_21978395_21978591_+.pdf]

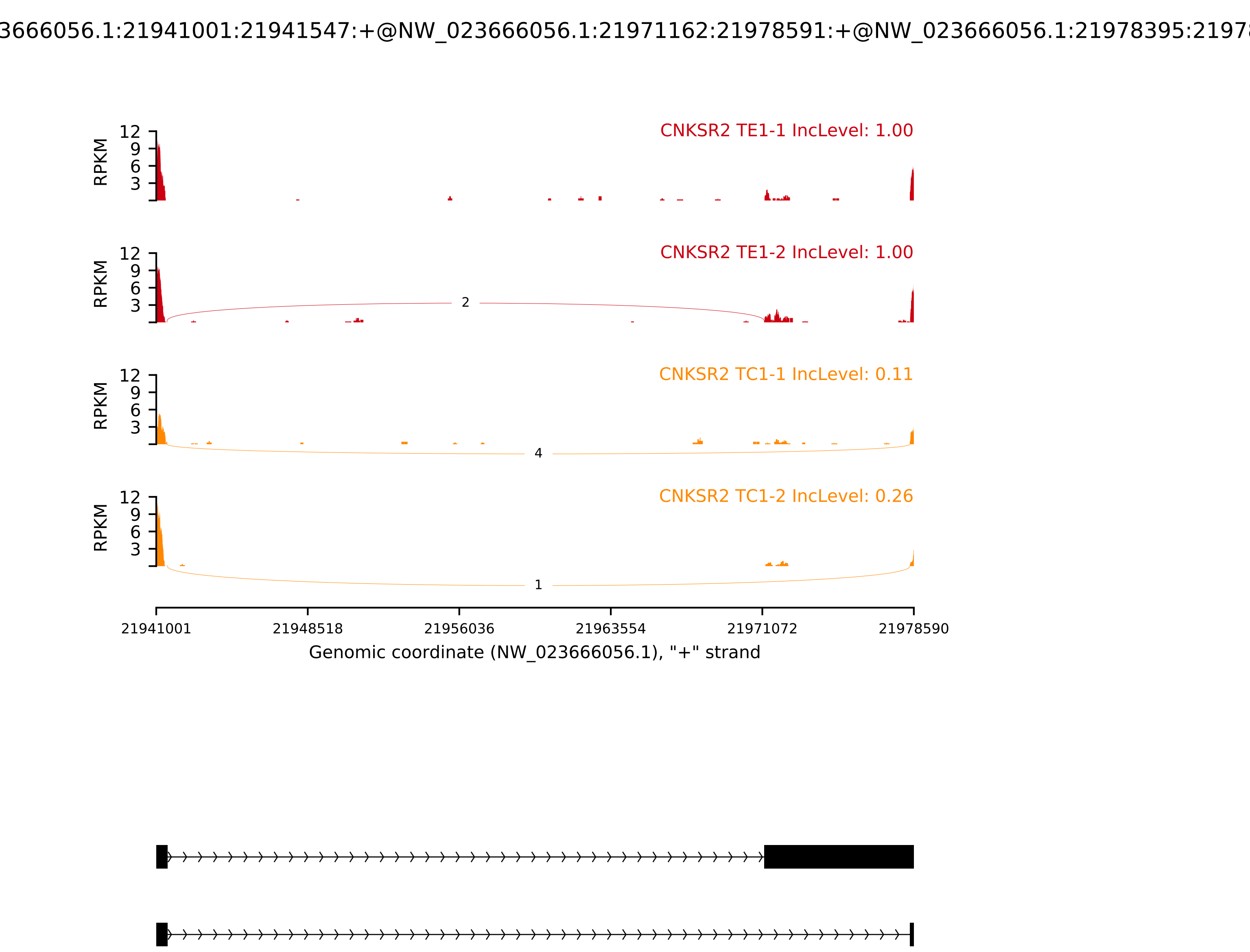
3666056.1:21941001:21941547:+@NW_023666056.1:21971162:21978591:+@NW_023666056.1:21978395:2197
RPKM
12
9
6
3
CNKSR2 TE1-1 IncLevel: 1.00
CNKSR2 TE1-2 IncLevel: 1.00
2
CNKSR2 TC1-1 IncLevel: 0.11
4
CNKSR2 TC1-2 IncLevel: 0.26
1
21941001
21948518
21956036
21963554
21971072
21978590
Genomic coordinate (NW_023666056.1), "+" strand
